# Frequent loss of the *AXIN1* locus but absence *of AXIN1* gene mutations in adenocarcinomas of the gastro-oesophageal junction with nuclear *β*-catenin expression

**DOI:** 10.1038/sj.bjc.6601589

**Published:** 2004-02-17

**Authors:** L B Koppert, A W van der Velden, M van de Wetering, M Abbou, A M W van den Ouweland, H W Tilanus, B P L Wijnhoven, W N M Dinjens

**Affiliations:** 1 Department of Pathology, Josephine Nefkens Institute, Erasmus University Medical Center, Rotterdam, The Netherlands; 2 Department of Surgery, Erasmus University Medical Center, Rotterdam, The Netherlands; 3 Hubrecht Laboratory, Center for Biomedical Genetics, Utrecht, The Netherlands; 4 Department of Clinical Genetics, Erasmus University Medical Center, Rotterdam, The Netherlands

**Keywords:** oesophageal cancer, Wnt signalling pathway, *AXIN1*, mutation, loss of heterozygosity

## Abstract

Up to 60% of gastro-oesophageal junction (GEJ) adenocarcinomas show nuclear *β*-catenin expression, pointing to activated T-cell factor (TCF)/*β*-catenin-driven gene transcription. We demonstrate in five human GEJ adenocarcinoma cell lines that nuclear *β*-catenin expression indeed correlates with enhanced TCF-mediated transcription of a reporter gene. In several tumour types, TCF/*β*-catenin activation is caused by mutations in either adenomatous polyposis coli (*APC*), *β*-*catenin* exon 3, *AXIN1*, *AXIN2* or *β*-transducin repeat-containing protein (*β-TrCP*). In GEJ adenocarcinomas, very few *APC* and *β*-*catenin* mutations have been found. Therefore, the mechanism of Wnt pathway activation remains unclear. In the present study, we did not find *AXIN1* gene mutations in 17 GEJ tumours with nuclear *β*-catenin expression (without *β*-*catenin* exon 3 mutations). Six intragenic single nucleotide polymorphisms (SNPs) were identified. One of these, the *AXIN1* gene T1942C SNP, has a frequency of 21% but is only very recently described despite numerous *AXIN1* gene mutational studies. We provide evidence why this SNP was missed in single strand conformation polymorphism analyses. The *AXIN1* gene G2063A variation was previously described as a gene mutation but we demonstrate that this is a polymorphism. With these six SNPs loss of heterozygosity (LOH) was found in 11 of 15 (73%) informative tumours. To investigate a possible *AXIN1* gene dosage effect in GEJ tumours expressing nuclear *β*-catenin, *AXIN1* locus LOH was determined in 20 tumours expressing membranous and no nuclear *β*-catenin. LOH was found in 10 of 13 (77%) informative cases. AXIN1 protein immunohistochemistry revealed cytoplasmic expression in all tumours irrespective of the presence of *AXIN1* locus LOH. These data indicate that nuclear *β*-catenin expression is indicative for activated Wnt signalling and that neither *AXIN1* gene mutations nor *AXIN1* locus LOH are involved in Wnt pathway activation in GEJ adenocarcinomas.

The incidence of adenocarcinoma of the gastro-oesophageal junction (GEJ), that is, distal oesophagus and gastric cardia, is rising in the Western world (Blot *et al*, 1991; Powell and McConkey, 1992; Botterweck *et al*, 2000). Patients with GEJ adenocarcinoma have a poor prognosis with 5-year survival rates of less than 25% (Tilanus *et al*, 1993). Despite the common occurrence of this malignancy, relatively little is known about the molecular mechanisms underlying the genesis and the progression of these tumours.

Numerous studies focused on cell–cell adhesion in GEJ adenocarcinomas since defective cell–cell adhesion is an important feature in epithelial tumour initiation and progression (Jankowski *et al*, 1997; Hirohashi, 1998; Nollet *et al*, 1999; Wijnhoven *et al*, 2000a; Van Aken *et al*, 2001; Hajra and Fearon, 2002). Aberrant expression of components of the E-cadherin–catenin complex, the prime mediator of epithelial cell–cell adhesion, has been found frequently in GEJ adenocarcinomas. In addition, E-cadherin–catenin complex aberrations appeared to have prognostic value in these tumours (Jian *et al*, 1997; Krishnadath *et al*, 1997; Bailey *et al*, 1998; Washington *et al*, 1998; Bian *et al*, 2000; Osterheld *et al*, 2002).

Recently, the E-cadherin–catenin complex component *β*-catenin has been demonstrated to play a dual role in the tumorigenic process (Korinek *et al*, 1997; Morin *et al*, 1997; Resnik, 1997; Rubinfeld *et al*, 1997; Bienz and Clevers, 2000; Polakis, 2000). *β*-Catenin, originally described to function in epithelial cell–cell adhesion, is implicated also in the Wnt signalling cascade as a transcriptional activator. In the absence of Wnt signals, *β*-catenin is located at the plasma membrane, linked to E-cadherin, and functions in cell–cell adhesion. Excess cytoplasmic *β*-catenin is sequestered in a protein complex comprised of adenomatous polyposis coli (APC), glycogen synthase kinase 3*β* (GSK-3*β*) and AXIN1 or AXIN2. In this complex, *β*-catenin is phosphorylated by GSK-3*β* and then targeted by *β*-transducin repeat-containing protein (*β*-TrCP) to proteasomal degradation. Activated Wnt signalling inhibits the phosphorylation of *β*-catenin, thereby preventing its degradation. The impaired *β*-catenin degradation leads to an increase in cytoplasmic *β*-catenin and its translocation to the nucleus. Nuclear *β*-catenin forms heterodimers with members of the T-cell factor (TCF) family of transcription factors and activates genes containing TCP-binding sites (Bienz and Clevers, 2000; Polakis, 2000).

It has been demonstrated that *β*-catenin/TCF-activated gene transcription can induce neoplastic transformation (Aoki *et al*, 1999; Kolligs *et al*, 2002; Miyoshi *et al*, 2002), and the *β*-catenin/TCF target genes comprise the oncogenes *c-myc* (He *et al*, 1998), *cyclin* D1 (Tetsu & McCormick, 1999) and *ITF*-2 (Kolligs *et al*, 2002). Impaired degradation of *β*-catenin in tumours has been reported to be caused by inactivating mutations in *APC*, *AXIN1*, *AXIN2* or *β-TrCP* or oncogenic mutations in *β-catenin* exon 3 (Kinzler and Vogelstein, 1996; Bienz and Clevers, 2000; Liu *et al*, 2000; Polakis, 2000; Satoh *et al*, 2000b; Gerstein *et al*, 2002; Hajra and Fearon, 2002; Taniguchi *et al*, 2002; Yokota *et al*, 2002). These mutations are in most cases mutually exclusive (Sparks *et al*, 1998; Huang *et al*, 2000; Gayet *et al*, 2001; Gerstein *et al*, 2002; Taniguchi *et al*, 2002; Yokota *et al*, 2002).

Several studies have reported nuclear *β*-catenin expression in up to 60% of GEJ adenocarcinomas (Bailey *et al*, 1998; Washington *et al*, 1998; Bian *et al*, 2000; Wijnhoven *et al*, 2000b; Osterheld *et al*, 2002). This nuclear expression of *β*-catenin can be regarded as an indication for activated, oncogenic, *β*-catenin/TCF transcription. However, mutation analysis of *APC* and *β-catenin* in GEJ adenocarcinomas revealed mutations in only less than 7 and 3% of cases respectively (Powell *et al*, 1994; Gonzalez *et al*, 1997; Bian *et al*, 2000; Choi *et al*, 2000). In accordance with these data, we recently did not find *β-catenin* exon 3 mutations in a series of 69 GEJ adenocarcinomas (Wijnhoven *et al*, 2000b). Inactivation of the *AXIN1* gene has been demonstrated to induce *β*-catenin/TCF transcription, and *AXIN1* gene mutations have been described in hepatocellular carcinomas, hepatoblastomas, colorectal cancers, ovarian endometrioid adenocarcinomas and in sporadic medulloblastomas (Satoh *et al*, 2000b; Webster *et al*, 2000a; Laurent-Puig *et al*, 2001; Dahmen *et al*, 2001b; Wu *et al*, 2001; Shimizu *et al*, 2002; Taniguchi *et al*, 2002; Yokota *et al*, 2002; Baeza *et al*, 2003; Miao *et al*, 2003). In addition, reduced protein expression of AXIN1 has recently been reported to correlate with tumour progression in oesophageal squamous cell carcinoma (Nakajima *et al*, 2003). These results prompted us to search for genomic aberrations in the *AXIN1* gene in GEJ adenocarcinomas. From the previously investigated series of 69 GEJ adenocarcinomas, 17 tumours with prominent nuclear *β*-catenin expression were selected for mutation and loss of heterozygosity (LOH) analysis. The entire coding region including the exon–intron boundaries of the *AXIN1* gene was analysed for genetic alterations by single strand conformation polymorphism (SSCP) analysis. The presence of six intragenic single nucleotide polymorphisms (SNPs) was used to detect LOH. These six SNPs were also used to perform *AXIN1* gene LOH analysis in 20 tumours with strong membranous *β*-catenin expression. In addition, AXIN1 protein expression was investigated by immunohistochemistry in all 37 tumour samples.

## MATERIALS AND METHODS

### TCF/*β-*catenin reporter gene assay

All cell lines, JROECL19, JROEL33, SKGT-4, TE-7, OACP4C and SW480, were cultured in RPMI 1640 supplemented with 10% fetal calf serum and antibiotics. Transcriptional activation mediated by TCF/*β*-catenin protein complexes was determined by transient transfection of the cell lines with either the pTOPGLOW or pFOPGLOW reporter constructs as described previously (van de Wetering *et al*, 2001). The pTOPGLOW and pFOPGLOW constructs contain a multimerized wild-type or mutant TCF binding motive, respectively, upstream of a luciferase gene. Cells were grown to 50–80% confluency in six-well plates and transfection was performed with 1 *μ*g of purified constructs each, using Fugene-6 (Boehringer, Mannheim, Germany). Transfection efficiencies were determined by cotransfection of a pRL-TK reporter construct (Promega, Madison, WI, USA) that contained the *Renilla* luciferase gene under control of the herpes simplex virus thymidine kinase promoter. Cells were harvested 24 h after transfection. Activity of both luciferases was measured sequentially in each sample using the Dual-Luciferase Reporter Assay System (Promega, Madison, WI, USA). TCF-mediated gene transcription was defined by the ratio of pTOPGLOW to pFOPGLOW luciferase activities. The luciferase activity of the internal control reporter was used to correct for differences in transfection efficiency.

### Tumour and cell line DNA samples

In a previous study of 69 GEJ adenocarcinoma samples consisting of 54 primary tumours, four lymph node metastases, nine xenografts and two *in vitro* cell lines were investigated for *β*-catenin expression and *β*-catenin exon 3 mutations (Wijnhoven *et al*, 2000b). From all cases, tumour and normal DNA was isolated from frozen samples by standard proteinase K digestion and phenol/chloroform extraction. After *β*-catenin immunohistochemistry on 5 *μ*m paraffin sections, parts of the tumour with intense nuclear reactivity were isolated by microdissection from consecutive unstained sections. From the microdissected fragments, DNA was isolated with standard proteinase K digestion followed by phenol/chloroform extraction and ethanol precipitation. No *β*-catenin exon 3 aberrations were found in these samples. For the present study, the DNA from 17 tumours with strong nuclear *β*-catenin expression was used. Fifteen samples were originated from primary GEJ adenocarcinomas and two were from GEJ adenocarcinoma-derived cell lines JROECL19 and JROECL33 (Rockett *et al*, 1997). In addition, the DNAs from 20 tumours with strong membranous *β*-catenin expression were investigated for LOH with the six SNPs. These 20 samples comprised 17 primary tumours and the established cell lines OACP4C, SKGT-4 and TE-7 (Altorki *et al*, 1993; Nishihira *et al*, 1993; de Both *et al*, 2001). Cell lines JROECL19 and 33 were obtained from the European Collection of Cell Cultures (ECACC, Wiltshire, UK), SKGT-4 and TE-7 were kind gifts from D Schrump, NIH, Bethesda, USA and T Kudo, Tohoku University, Sendai, Japan, respectively; OACP4C was established at our own institute. From cell lines JROECL19 and JROECL33, patient's normal tissue was kindly provided by Dr SJ Darnton, Birmingham Heartlands Hospital, Birmingham, UK. From cell line OACP4C patient's normal tissue was obtained from our pathology archive, and from cell lines SKGT-4 and TE-7 patient's normal tissue was not available. To determine SNP frequencies in the normal population, we used DNA isolated from 161 healthy Caucasian blood donor volunteers.

### Mutation analysis of the *AXIN1* gene

The 17 pairs of tumour and normal DNA from tumours with nuclear *β*-catenin expression were screened for aberrations in the *AXIN1* gene. The entire coding sequence, including the exon–intron boundaries, was investigated by PCR-SSCP using the previously described 23 sets of primers with slight modifications (Lin *et al*, 2000). All amplifications were performed in 15 *μ*l PCR containing 50–100 ng DNA, 1.5 mM MgCl_2_, 0.02 mM dATP, 0.2 mM dGTP, dCTP and dTTP each, 0.8 *μ*Ci of [^32^P]dATP (Amersham Biosciences, Buckinghamshire, UK), 20 pmol of each primer and 0.3 U AmpliTaq Gold polymerase (Perkin-Elmer Applied Biosystems, Foster City, CA, USA). AmpliTaq Gold polymerase was used because this enzyme is superior to other DNA polymerases with regard to amplification of DNA retrieved from routine formalin-fixed and paraffin-embedded tissues. To the PCRs with primer sets 4, 10, 12, 13, 14, 15, 18, 21 and 23, DMSO (5%) was added to increase the amplification efficiency. The PCRs were performed with a hotstart of 95°C for 5min followed by 35 cycles of 95°C for 30 s, 55°C (primer sets 10 and 14 at 58 and 60°C, respectively) for 45 s and 72°C for 45s and ended up with an extension step of 72°C for 10 min. The PCR products were diluted 1 : 4 with loading buffer (95% formamide, 10 mM EDTA, 0.05% bromophenol blue and 0.05% xylene cyanol), heated for 5 min, cooled on ice and electrophoresed in 6% polyacrylamide gels containing 10% glycerol at 7 W overnight at room temperature, in 1 × TBE running buffer. Gels were dried and exposed to X-ray films at −80°C. Results were evaluated by visual inspection. With the used SSCP conditions, the PCR products from primer sets 18, 19 and 21 resulted in simple banding patterns, inefficient for the detection of aberrations. To increase the DNA aberration detection efficiency, these PCR products were electrophoresed without glycerol at 4°C for 6 h resulting in more complex banding patterns. For each variant SSCP pattern identified by SSCP analysis, the genomic DNA samples were reamplified for bidirectional direct sequencing with the amplification primers. In 20 DNA samples from tumours with strong membranous *β*-catenin expression, LOH was determined by SSCP analysis of six detected SNPs. These SNPs are at positions (according to GenBank accession no. AF009674) A94C, C874T, intron 4+17 G → A (nucleotide position identified from exon–intron boundary), G1396A, T1942C and G2063A. Amplification of these SNPs was performed with primer sets 1, 7, 11, 12 and 17, respectively.

### *β*-Catenin and AXIN1 immunohistochemistry

In all 37 tumour samples, immunostaining for *β*-catenin and AXIN1 was performed on 5 *μ*m paraffin sections with a mouse anti-human *β*-catenin monoclonal antibody (Transduction Laboratories, Lexington, KY, USA; 1 : 200, 30 min, room temperature) (Wijnhoven *et al*, 2000b) and a rabbit polyclonal anti-human AXIN1 antibody (Zymed Laboratories, San Francisco, CA, USA; 1 : 25, 30 min, room temperature), respectively. After deparaffinisation and treatment with methanol/H_2_O_2_, antigen retrieval was performed in citrate buffer for 15 min prior to incubation with the *β*-catenin antibody. No antigen retrieval was necessary for the AXIN1 immunohistochemistry. Immunoreactivity was made visible by a standard avidin biotin immunoperoxidase technique, using a commercially available kit (Labvision, Fremont, CA, USA) and diaminobenzidine hydrochloride (Fluka, Neu-Ulm, Germany). As negative controls, normal mouse immunoglobulins and normal rabbit serum were used.

## RESULTS

### TCF/*β*-catenin reporter gene assay

**Figure 1:**
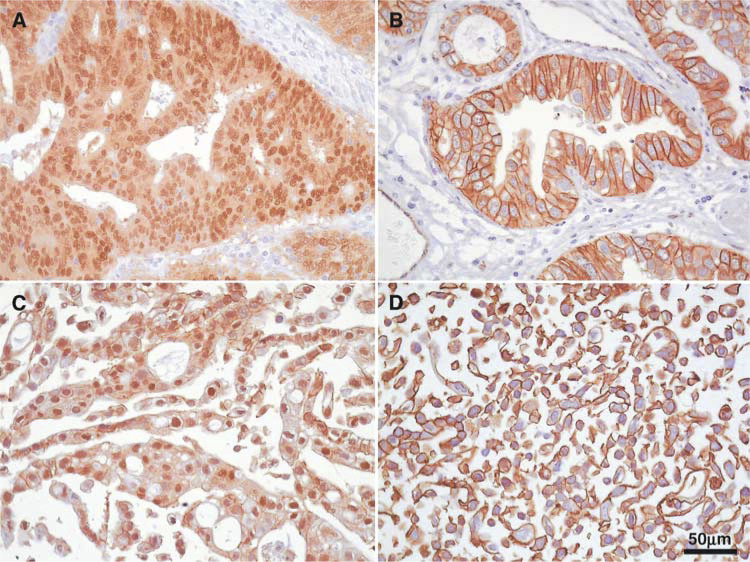
Immunohistochemistry of *β*-catenin in GEJ adenocarcinomas and cell lines (*β*-catenin antibody, DAB and haematoxylin counterstain, magnification × 400). (**A**) GEJ adenocarcinoma. Strong nuclear expression of *β*-catenin in the tumour cells. (**B**) GEJ adenocarcinoma. Prominent membranous expression of *β*-catenin. (**C**) Cell line JROECL19. Strong nuclear expression of *β*-catenin. (**D**) Cell line TE-7. Membranous expression of *β*-catenin.

**Figure 2:**
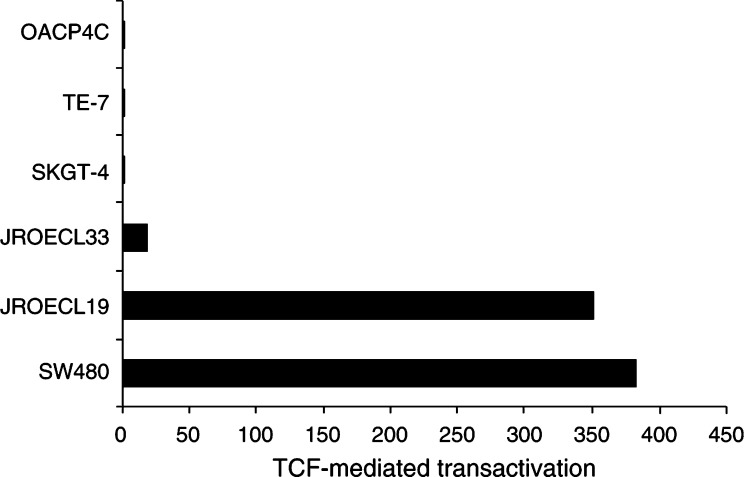
TCF-mediated transcriptional activation in GEJ adenocarcinoma cell lines. Constitutive transcriptional activation was detected in cell lines JROECL33 and JROECL19. APC mutant colorectal cancer cell line SW480 served as a positive control. TCF-mediated transcriptional activity was defined as the ratio of pTOPGLOW : pFOPGLOW luciferase activities, each corrected for pRL-TK luciferase activities and where no transactivation equals 1.

The TCF/*β*-catenin reporter gene assay was performed with the pTOPGLOW and pFOPGLOW constructs. Cell lines JROECL19 and JROECL33, both with nuclear *β*-catenin expression (JROECL19, Figure 1C

), showed 350- and 18-fold increase in transcriptional activity of the pTOPGLOW reporter as compared to the negative control pFOPGLOW (Figure 2

). Cell lines SKGT-4, TE-7 and OACP4C, all with membranous *β*-catenin immunoreactivity (TE-7, Figure 1D), showed no enhanced transcription of the pTOPGLOW reporter (Figure 2).

### Mutation analysis of the *AXIN1* gene

**Figure 3:**
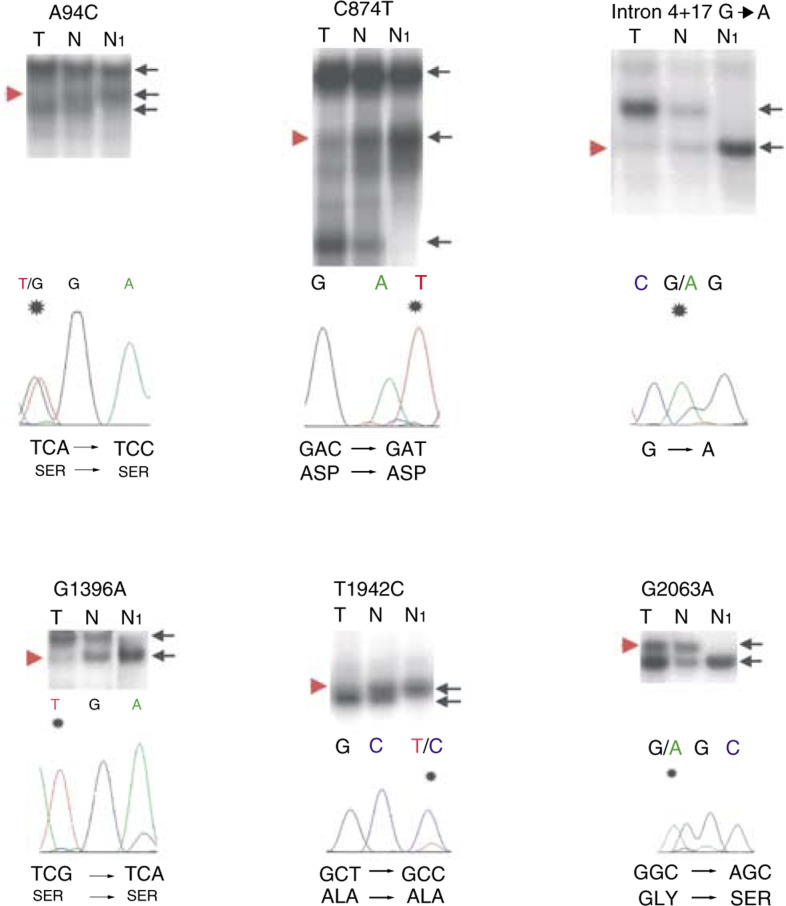
PCR-SSCP and sequencing analyses of the SNPs in tumours (T) and corresponding normal DNA (N), compared with DNA from individuals without SNPs (N1). Informative cases with LOH are shown. Black arrows point to allelic patterns. Red arrow heads point to deleted alleles in the tumour DNA. The sequencing chromatograms below each autoradiograph show the alterations (note the substituted nucleotide marked by an asterisk), which all represent SNPs. SNPs A94C and G1396A are annotated in the reverse complementary direction, whereas the SNPs C874T, intron 4+17 G → A, T1942C and G2063A are annotated in the forward direction.

SSCP analysis of the *AXIN1* gene in 17 tumour/normal DNA pairs from tumours with strong nuclear *β*-catenin expression (Figure 1A, C) revealed six different aberration patterns. All these aberrations were also present in the corresponding normal DNA. Sequencing of the aberrant samples identified six different SNPs. These were at positions (according to GenBank accession no. AF009674) A94C, C874T, intron 4+17 G → A, G1396A, T1942C and G2063A (Figure 3

).

Of the 17 cases, 15 appeared to be heterozygous for at least one polymorphism and LOH was observed in 11 (73%) cases (Table 1

**Table 1:** Patterns of allelic loss in GEJ adenocarcinomas with nuclear ß-catenin expression

**Case**	**A94C**	**C874T**	**Intron 4+17 G → A**	**G1396A**	**T1942C**	**G2063A**
1	Loss	HN	Loss	HN	HN	HN
2	HN	No loss	HN	No loss	Loss	HN
3	HN	HN	HN	HN	HN	HN
4	HP	HN	Loss	HN	HN	HN
5	Loss	HN	HN	HN	HN	HN
6	HN	HP	HN	No loss	HN	HN
7	HN	HP	HN	No loss	HN	HN
8	Loss	HN	Loss	No loss	No loss	HN
9	HN	Loss	HN	HN	HN	HN
10	No loss	HN	No loss	No loss	HN	HN
11	HN	HN	HN	HN	HN	HN
12	HN	HN	HN	HN	HN	HN
13	HN	HN	HN	Loss	Loss	HN
14	HN	HN	HN	Loss	HN	HN
15	HN	Loss	HN	Loss	HN	HN
Frequency	6/34 (18%)	8/34 (24%)	4/34 (12%)	9/34 (26%)	4/34 (12%)	1/34 (3%)
Frequency NP	ND	ND	ND	ND	69/322 (21%)	9/322 (3%)

HN=homozygous normal; HP=homozygous polymorphism; NP=normal population; ND=no data.

). The polymorphisms were used to investigate LOH in 20 GEJ adenocarcinoma cases with strong membranous *β*-catenin expression (Figure 1B, D). Also in this series, all six polymorphisms were present and in 10 of 13 (77%) informative cases LOH was found (Table 2

**Table 2:** Patterns of allelic loss in GEJ adenocarcinomas without nuclear ß-catenin expression

**Case**	**A94C**	**C874T**	**Intron 4+17 G → A**	**G1396A**	**T1942C**	**G2063A**
1	No loss	HP	Loss	HN	Loss	HN
2	HN	Loss	HN	HN	Loss	HN
3	HN	HN	HN	Loss	Loss	HN
4	HN	HN	HN	HN	Loss	HN
5	HN	HP	HN	HN	Loss	HN
6	HN	HP	HN	Loss	HN	Loss
7	HN	No loss	HN	HN	HN	HN
8	No loss	HP	No loss	HN	HN	HN
9	HN	HN	HN	HN	HN	HN
10	HN	HN	HN	HN	HN	HN
11	Loss	HP	Loss	HN	HN	HN
12	Loss	Loss	HN	Loss	Loss	HN
13	HN	HP	HN	HN	HP	HN
14	HN	HN	HN	HN	HN	HN
15	HP	HP	HP	HN	HN	HN
16	No loss	HP	No loss	HN	No loss	HN
17	HN	Loss	HN	HN	HN	HN
Frequency	7/40 (18%)	20/40 (50%)	6/40 (15%)	5/40 (13%)	9/40 (23%)	2/40 (5%)
Frequency NP	ND	ND	ND	ND	69/322 (21%)	9/322 (3%)

For abbreviations see Table 1 footnote.

).

**Figure 4:**
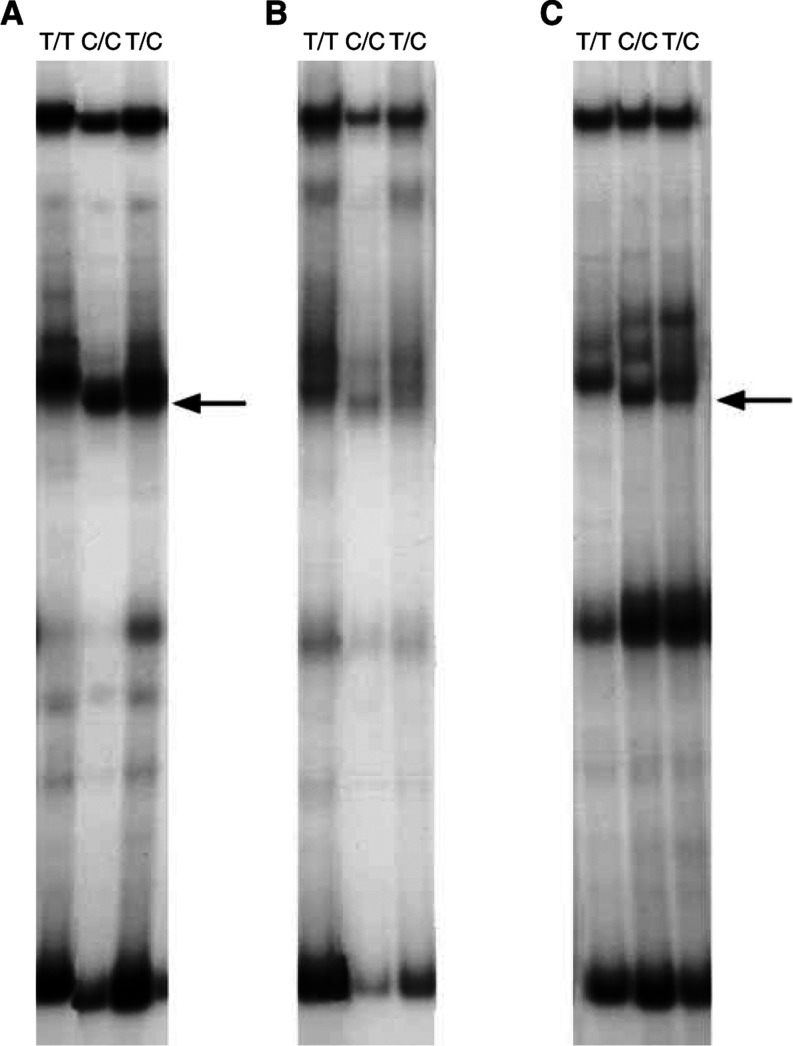
T1942C PCR-SSCP. Three DNA samples, 1942T/T (homozygous normal), 1942C/C (homozygous polymorphic), 1942T/C (heterozygous), were amplified with AmpliTaq Gold polymerase and AmpliTaq Gold buffer (**A**), Promega Taq and Promega buffer (**B**), Promega Taq and AmpliTaq Gold buffer (**C**) and with AmpliTaq Gold and Promega buffer (no PCR products obtained). All samples were amplified and SSCP-electrophoresed in the same experiment. Arrows point to the polymorphic SSCP fragment. Note that the polymorphic fragments are clearly visible only after amplification in AmpliTaq Gold buffer (**A**, **C**).

The T1942C and G2063A SNPs were found to have population frequencies of 21 and 3%, respectively (Tables 1 and 2). The relatively frequent SNP T1942C was only described very recently in two independent studies (Baeza *et al*, 2003; Miao *et al*, 2003) and remained undetected in 10 *AXIN1* gene mutation studies (Lin *et al*, 2000; Satoh *et al*, 2000a; Webster *et al*, 2000b; Dahmen *et al*, 2001a; Wu *et al*, 2001; Miao *et al*, 2002; Shimizu *et al*, 2002; Taniguchi *et al*, 2002; Yokota *et al*, 2002; Nakajima *et al*, 2003). In most of these studies, as in ours, SSCP analyses were used. To determine whether the SSCP conditions have influence on the detection of the T1942C SNP, we amplified DNA samples with AmpliTaq Gold and with Promega Taq DNA polymerases (Promega, Madison, WI, USA) in both AmpliTaq Gold and Promega Taq buffers. The AmpliTaq Gold buffer consists of 15 mM Tris-HCl (pH 8.0), 50 mM KCl and 1.5 mM MgCl_2_ and the Promega buffer consists of 10 mM Tris–HCl (pH 9.0), 50 mM KCl, 1.5 mM MgCl_2_ and 0.1% Triton X-100. As demonstrated in Figure 4

, the T1942C SNP is only clearly visible after amplification with the AmpliTaq buffer.

### *β*-Catenin and AXIN1 immunohistochemistry

**Figure 5:**
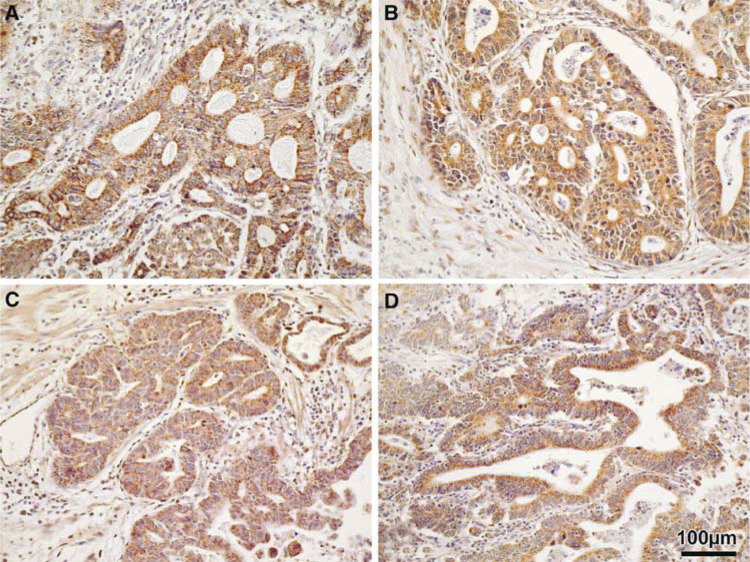
Immunohistochemistry of AXIN1 in GEJ adenocarcinomas (AXIN1 antibody, DAB and haematoxylin counterstain, magnification × 200). (**A**, **B**) GEJ adenocarcinomas with nuclear *β*-catenin expression without (**A**) and with (**B**) *AXIN1* locus LOH. (**C**, **D**) GEJ adenocarcinomas with membranous *β*-catenin expression without (**C**) and with (**D**) *AXIN1* locus LOH. Note the strong cytoplasmic AXIN1 expression in the tumour cells in all four cases.

As mentioned before, 37 GEJ adenocarcinoma samples were investigated, 17 with strong nuclear *β*-catenin expression and 20 cases with normal membranous reactivity (Figure 1). Eleven of the 15 (73%) informative cases with nuclear *β*-catenin expression and 10 of 13 (77%) informative cases with membranous *β*-catenin immunoreactivity showed loss of the *AXIN1* gene locus. In all tumour samples AXIN1 protein expression was confined to the cytoplasm of the tumour cells exclusively (Figure 5

). No consistent differences in AXIN1 protein expression were observed between tumours with and without *AXIN1* locus loss, irrespective of nuclear or membranous *β*-catenin expression (Figure 5).

## DISCUSSION

Nuclear *β*-catenin expression has been reported in up to 60% of GEJ adenocarcinomas (Bailey *et al*, 1998; Washington *et al*, 1998; Bian *et al*, 2000; Wijnhoven *et al*, 2000b; Osterheld *et al*, 2002). In the present study, we used the nuclear expression of *β*-catenin in GEJ adenocarcinomas as an indication for activated Wnt signalling. This presupposition is substantiated by our finding of highly enhanced TCF-mediated transcriptional activity in the cell lines JROECL19 and JROECL33, both with nuclear *β*-catenin expression. None of the three cell lines without nuclear but with membranous *β*-catenin expression had increased transcription of the TOPGLOW reporter. Activated Wnt signalling in tumours is caused by increased levels of *β*-catenin, which can be the result of mutations in *APC*, *β-catenin, AXIN1, AXIN2* or *β-TrCP* (Kinzler and Vogelstein, 1996; Bienz and Clevers, 2000; Liu *et al*, 2000; Polakis, 2000; Satoh *et al*, 2000b; Gerstein *et al*, 2002; Hajra and Fearon, 2002; Taniguchi *et al*, 2002; Yokota *et al*, 2002). To date only *APC* and *β-catenin* mutation analysis in GEJ adenocarcinomas has been performed and only few mutations were found (Powell *et al*, 1994; Gonzalez *et al*, 1997; Bian *et al*, 2000; Choi *et al*, 2000; Wijnhoven *et al*, 2000b). Therefore, the mechanism of Wnt activation remains obscure in these tumours. These results prompted us to investigate GEJ adenocarcinoma samples with nuclear *β*-catenin expression for mutations in the *AXIN1* gene. None of the tumours in the present study showed *β-catenin* exon 3 mutations (Wijnhoven *et al*, 2000b). In 17 GEJ adenocarcinoma samples, no *AXIN1* gene mutations were found. We therefore conclude that Wnt activation in GEJ tumours is not caused by *AXIN1* gene mutations.

Six previously described polymorphisms in the *AXIN1* gene were detected. All the detected DNA variations were also found in patients' constitutional DNAs indicating that they are truly polymorphisms and not somatic mutations. The G2063A SNP in exon 6 results in a substitution from glycine to serine. This polymorphism is described by Webster *et al* (2000a), although these authors annotated the polymorphism to exon 7 and regarded it as a silent polymorphism. Furthermore, in a recent study by Taniguchi *et al* (2002), the G2063A polymorphism is found in one hepatocellular carcinoma and in one hepatoblastoma. Patients' normal tissues were not investigated and because the SNP was not found in their 147 control individuals, they regarded it as an *AXIN1* mutation. We found a G2063A allele frequency of 4 and 3% in GEJ adenocarcinoma patients and healthy controls, respectively, indicating that this *AXIN1* gene variant is an SNP and not a mutation. In addition, we found a silent T1942C SNP (ala-ala) in exon 6 with an allele frequency of 18 and 21% in GEJ adenocarcinoma patients and healthy controls, respectively. This frequent SNP was only recently described, with comparable allele frequencies, in two independent studies (Baeza *et al*, 2003; Miao *et al*, 2003) and remained undetected in 10 *AXIN1* gene mutation investigations within total 670 DNA samples (Lin *et al*, 2000; Satoh *et al*, 2000b; Webster *et al*, 2000b; Dahmen *et al*, 2001a; Wu *et al*, 2001; Miao *et al*, 2002; Shimizu *et al*, 2002; Taniguchi *et al*, 2002; Yokota *et al*, 2002; Nakajima *et al*, 2003). We demonstrate that detection of the T1942C SNP by SSCP analysis is influenced by the PCR buffer characteristics. This finding suggests that also other SSCP parameters (gel composition, running buffer, running temperature, etc) can have influence on the SNP detection.

The *AXIN1* gene polymorphisms were used to determine LOH. Fifteen of 17 cases with nuclear *β*-catenin expression appeared to be heterozygous for at least one polymorphism, and from these in 11 (73%) clear LOH was observed. This frequent loss of the *AXIN1* gene could point to a dosage effect where the presence of 50% of the AXIN1 protein is insufficient for proper *β*-catenin degradation and subsequently leads to Wnt activation. To gather more information about this possibility, we investigated *AXIN1* gene LOH with the detected SNPs in a series of 20 tumours from our previous study with strong, normal membranous *β*-catenin expression. These included three cell lines (OACP4C, SKGT-4 and TE-7) without enhanced TCF-mediated transcriptional activity. In this series, the T1942C and G2063A SNPs were detected in four and two samples, respectively. A total of 13 cases were informative and in 10 (77%) clear LOH was observed.

By AXIN1 immunohistochemistry, no differences were observed between tumours with and without *AXIN1* locus loss. It is known that with the semiquantitative immunohistochemical method a two-fold reduction in protein expression cannot be detected. In oesophageal squamous cell carcinoma, reduced protein and RNA expression has recently been described by Nakajima *et al* (2003). These investigators suggest *AXIN1* gene silencing by promotor methylation, in addition to allelic losses, as a mechanism for AXIN1 downregulation. Our immunohistochemical results indicate that *AXIN1* gene silencing does not occur in GEJ adenocarcinomas.

All the above-mentioned results indicate that *AXIN1* gene haplo-insufficiency is not sufficient for Wnt activation. This is in accordance with studies that demonstrated biallelic inactivation of the *AXIN1* gene in several tumour types (Satoh *et al*, 2000b; Laurent-Puig *et al*, 2001; Taniguchi *et al*, 2002; Yokota *et al*, 2002), although heterozygous *AXIN1* gene inactivation has also been described (Dahmen *et al*, 2001b; Webster *et al*, 2000a). Chromosome 16p loss has been reported in up to 40% in GEJ adenocarcinomas (Gleeson *et al*, 1998) and the presence of a tumour suppressor gene on 16p13.3 has been suggested by Takamochi *et al* (2001). The frequent *AXIN1* locus LOH in our study could point to a gene dosage effect independent of Wnt pathway activation. Furthermore, it cannot be excluded that an as yet to be defined tumour suppressor gene on 16p13.3 is the actual target for LOH in GEJ adenocarcinomas.

Since mutations in *APC, β-catenin* and *AXIN1* do not play a major role in the frequent TCF/*β*-catenin activation in GEJ adenocarcinomas, other components should be considered. *AXIN2* gene mutations have been described in 11 colorectal cancers (Liu *et al*, 2000), one endometrioid ovarian adenocarcinoma (Wu *et al*, 2001) and two hepatocellular carcinomas (Taniguchi *et al*, 2002). Of these 14 tumour samples, 12 had nuclear *β*-catenin expression indicating activated Wnt signalling. The *AXIN2* gene mutations appear to be present exclusively in tumours with a microsatellite instable phenotype since all *AXIN2* mutant colorectal carcinomas and the ovarian endometrioid adencarcinoma were microsatellite instable (Wu *et al*, 2001; Taniguchi *et al*, 2002). The *AXIN2* gene is an improbable candidate for Wnt activation in GEJ adenocarcinomas because microsatellite instability has been described in only less than 6% in these tumours (Gleeson *et al*, 1996; Wijnhoven *et al*, 2000b). Another candidate is *β-TrCP*, involved in *β*-catenin degradation. Recently, *β-TrCP* mutations have been described in two prostate cancer samples, of which one had nuclear *β*-catenin expression (Gerstein *et al*, 2002).

Activated Wnt signalling can also be the result of activation of the canonical Wnt pathway by secreted Wnts or by expression of the Wnt receptors frizzled (Tanaka *et al*, 1998; Mizushima *et al*, 2002). In cell line cultures, this would imply the presence of an autocrine Wnt/frizzled loop. Furthermore, tumour necrosis factor-*α* has recently been demonstrated to induce TCF/*β*-catenin-mediated transcription in a GEJ adenocarcinoma cell line (Tselepis *et al*, 2002). These findings indicate that next to mutational activation of the Wnt pathway, gene expression alterations should be considered also as driving force behind Wnt activation in GEJ adenocarcinomas.

In summary, our results demonstrate that nuclear *β*-catenin expression in GEJ adenocarcinoma cell lines correlates with TCF-mediated transcription activation and so with activated Wnt signalling. The frequent nuclear localisation of *β*-catenin in GEJ adenocarcinomas cannot be attributed by *AXIN1* gene mutations. The mechanism of Wnt activation in these tumours remains to be established. In addition, the role of the frequent LOH of the *AXIN1* locus in GEJ adenocarcinomas deserves further investigation.
